# Gelseminum for Dilatation of Cervix

**Published:** 1877-01-20

**Authors:** 


*Gelseminum for Dilatation of Cervix.*—Dr. Agnew, in *Va. Med. Monthly*, states that Gelseminum proved efficacious in three obstinate cases of rigid cervix. He gives ten drops every ten minutes until thirty drops are taken.

